# A Multi-Method Approach for Impact Assessment of Some Heavy Metals on *Lactuca sativa* L.

**DOI:** 10.3390/molecules28020759

**Published:** 2023-01-12

**Authors:** Maria-Loredana Soran, Aura Nicoleta Sîrb, Ildiko Lung, Ocsana Opriş, Otilia Culicov, Adina Stegarescu, Pavel Nekhoroshkov, Delia-Maria Gligor

**Affiliations:** 1 National Institute for Research and Development of Isotopic and Molecular Technologies, 67-103 Donat, 400293 Cluj-Napoca, Romania; 2 Faculty of Environmental Science and Engineering, Babeș-Bolyai University, 30 Fântânele, 400294 Cluj-Napoca, Romania; 3 Joint Institute for Nuclear Research, 6 Joliot-Curie, 1419890 Dubna, Russia; 4 National Institute for Research and Development in Electrical Engineering ICPE-CA, 313 Splaiul Unirii, 030138 Bucharest, Romania

**Keywords:** lettuce, heavy metals, bioactive compounds, antioxidant capacity, elemental content

## Abstract

Heavy metals represent a large category of pollutants. Heavy metals are the focus of researchers around the world, mainly due to their harmful effects on plants. In this paper, the influence of copper, cadmium, manganese, nickel, zinc and lead, present in soil in different concentrations (below the permissible limit, the maximum permissible concentration and a concentration higher than the maximum permissible limit) on lettuce (*Lactuca sativa* L.) was evaluated. For this purpose, the authors analyzed the variation of photosynthetic pigments, total polyphenols, antioxidant activity and the elemental content in the studied plants. The experimental results showed that the variation of the content of biologically active compounds, elemental content and the antioxidant activity in the plants grown in contaminated soil, compared to the control plants, depends on the type and concentration of the metal added to the soil. The biggest decrease was recorded for plants grown in soil treated with Ni I (−42.38%) for chlorophyll a, Zn II (−32.92%) for chlorophyll b, Ni I (−40.46%) for carotenoids, Pb I (−40.95%) for polyphenols and Cu III (−29.42%) for DPPH. On the other hand, the largest increase regarding the amount of biologically active compounds was registered for Mn I (88.24%) in the case of the chlorophyll a, Mn I (65.56%) for chlorophyll b, Pb I (116.03%) for carotenoids, Ni III (1351.23%) for polyphenols and Ni III (1149.35%) for DPPH.

## 1. Introduction

Cultivated land contamination with heavy metals is becoming a major concern nowadays [1]. This interest is due to the high toxicity of heavy metals, their persistency and bioaccumulative behavior, which are a threat to natural ecosystems [2]. In general, environmental contamination with heavy metals is originated from two main sources: natural sources and anthropogenic activities [3]. The contamination from natural sources includes geologic materials, forest fires and volcanic outcrops [4]. Anthropogenic activities (mining, automobile exhausts, the use of fertilizers and insecticide, domestic waste) have been identified as principal contamination sources, which led to an increasing level of heavy metals in soil [5,6]. After entering the environment, traces of metals can be retained in soil systems for a very long period of time because they are not biochemically degraded [7]. 

The accumulation of the metals in soil frequently disturbs the normal functioning of soil ecosystem and the growth of the plants [8]. Plants have the ability to absorb and accumulate various trace of heavy metals from the contaminated agricultural soils [9], chemical soil fertilizers, sewage irrigation, insecticides, pesticides and atmospheric particles deposition. The heavy metals are transported to different parts of the plant through different pathways, altering the physiological, metabolic and biochemical activities of the plant [10]. Currently many researches focus more and more on transferring heavy metals into edible parts of the plants (rice, wheat, vegetables) from farmlands irrigated with wastewaters [11,12,13,14]. 

Some metals, such as manganese (Mn), copper (Cu), zinc (Zn), molybdenum (Mo) and nickel (Ni), are crucial and useful micronutrients for microorganisms, plants and animals, but at high level concentrations these metals can have toxic effects and pose an environmental threat [15]. Among the heavy metals that cause harmful impacts to plants are chromium (Cr), cadmium (Cd) mercury (Hg), lead (Pb), Ni and Cu. These harmful metals are responsible for minimizing the profitability and damaging agro-biological systems [16]. Such soil pollutants are considered to be toxins for their extensive availability, and their intensity and persistency in soil. Each heavy metal exposes the plant to different ill-effects [17,18]. Heavy metals present in soil were demonstrated to have toxic effects on plants, leading to changes in physiological characteristics such as taproot length, plant height, leaf [1], nutrient homeostasis, photosynthesis, gas exchange characteristics, protein mobilization, enzyme and antioxidant production [19].

The permanent contact of the plants with higher toxic concentrations of Cd metal decreases photosynthesis, water and nutrient uptake by plants [20]. Cd is trivial and can have harmful effects for higher plants and other living beings [21]. It was observed that Cd has a negative influence on growth and development [22,23]. Additionally, the leaf water potential, stomatal conductance, transpiration and the total chlorophyll were reduced in plants exposed to Cd [24]. The growth of plants in contaminated soil with high levels of Cd leads to growth inhibition, chlorosis, and finally the death of the plant [20]. Grajek et al. [25] demonstrated that the degradation of chlorophyll by Cd is caused by the instantly replacement of magnesium (Mg) by Cd.

Mining and industrial activities, such as the melting of copper-containing ores, lead to the deposition of high quantities of Cu into the environment. This metal is considered to be an essential micronutrient for plants and has an important role in the assimilation of carbon and ATP synthesis [26]. Large quantities of Cu induces soil stress and damages plants causing development inhibition and chlorosis. Copper excess induced a concomitant increase in the expression of proteins involved in photosynthesis, respiration, and C, N and S assimilation in *Ulva compressa* [27]. A higher concentration of Cu exposed to plants produces oxidative pressure and ROS [28]. Important concentrations of Cu decreased the growth of *Scopelophila cataractae* [29], the chlorophyll content in tea plants [30,31] and the carotenoids from *Citrus aurantium* L. [31].

High levels of Zn metal in soil results from sewage sludge, the use of fertilizers, municipal waste emissions and anthropogenic activities. Enhanced levels of Zn from soil can cause several structural and functional abnormalities [32,33,34]. The important quantities of Zn in soil block many metabolic activities of the plant, restrict development and cause plant aging. Additionally, Zn affects the development of the root, shoots system and causes chlorosis, which can spread out other plant parts [21].

From human activities (mining, metallurgy, battery manufacturing, chemical catalysis) Mn is released into the environment and can cause health problems [35]. Even if Mn has a role in plant photosynthesis, enzymatic reactions, and redox activities [36,37], an excess of Mn quantity in soil inhibits plant growth and development [38]. Additionally, Mn when present in excess can causes disrupting photosynthesis and enzyme activity in plants [39]. In *Macleaya cordata* an excess level of Mn causes cells distortion and deformation, a decrease of mitochondria, the shrinkage of chloroplasts, an increase of hungry particles and a decrease of starch granules [40].

Ni is counted among the essential micro-elements for higher plant species but is necessary at very low concentrations. An excess of Ni content in soil can have a toxic effect to the plants, disturbing the development and growth of the plant, producing ROS, which strongly affects cell ultrastructure [41]. Additionally, an elevated level of Ni can cause stunted growth, chlorosis, nutrient imbalance, alterations of osmolytes or changes in enzyme activities [42]. The chlorophyll, carotenoid, and proline contents from *Vigna mungo* L. were seriously affected by Ni and Pb. Additionally, in the case of these two heavy metals, chlorophyll a was affected more in comparison to chlorophyll b, whereas carotenoids were less affected than chlorophylls [43].

Pb enters the soil from municipal sludges, paints, mining, gasoline and paper industries. Cenkci et al. [44] demonstrated that Pb has an impact on the development, morphology, and photosynthetic cycles of the plants. Additionally, Pb causes the inhibition of enzymes, membrane porosity changes, water disequilibrium, and the alteration of mineral supplements. Pb causes oxidative pressure expanding the ROS in plants [44,45] and inhibits the photosynthesis process [46]. Chlorophyll synthesis is inhibited by Pb, which leads to a reduced uptake of the essential elements, such as magnesium (Mg) and iron (Fe), by plants [47]. Pb treatments exposed to plants affects chlorophyll b more than chlorophyll a [48,49].

The novelty of this article consists of the evaluation of the influence of six heavy metals, with three different concentrations, on the bioactive compounds, antioxidant activity and the elemental content of lettuce, at the same time.

The aim of the present study was to investigate the influence of abiotic stress resulting from diverse heavy metals on lettuce (*Lactuca sativa* L.). Lettuce is a highly consumed vegetables [50] being rich in phenols, vitamin C, folic acid, carotenoids and chlorophyll, nutritional compounds which are beneficial for human health. Antioxidant micronutrients, such as polyphenols and carotenoids, play an essential role in preventive nutrition, human health and plant metabolism [51,52]. Due to high consumption and sensitivity to stress [53], in the present research, lettuce was chosen for the experimental material. Salts of six heavy metals (Cd, Cu, Zn, Mn, Ni and Pb), in different concentrations, were selected in the treatment of the lettuce in order to assess the impact on photosynthetic pigments (chlorophylls and carotenoids), total polyphenols, antioxidant activity and the variation of the multielement content.

## 2. Results

### 2.1. Analysis of Plant Tissues

#### 2.1.1. Assimilating Pigments Evaluation

**Figure 1:**
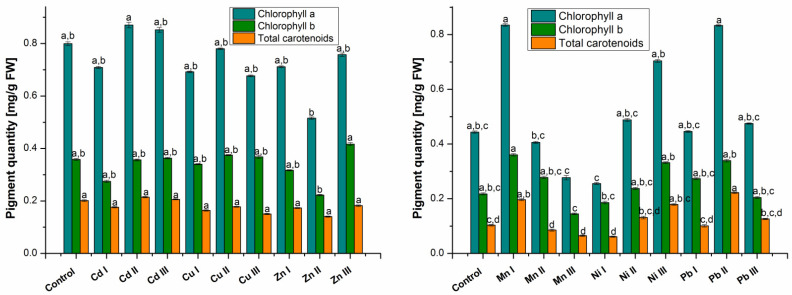
Comparative diagram of the pigment content. Each data point is the mean ± the standard error of the mean of three independent replicates experiments; different letters mean significant differences between the treatment and the control plants, determined by Tukey’s test (*p* < 0.05).

A decrease in the amount of chlorophylls and total carotenoids can be observed for plants grown in the presence of Cd I, Cu I, Zn I and Ni I, respectively (Figure 1). The amount of pigment that decreased the most was chlorophyll a (0.42 times Ni I), chlorophyll b (0.23 times Cd I) and total carotenoids (0.4 times Ni I and 0.19 times Cu I). In the case of Mn I, the amount of chlorophyll a (1.88 times), chlorophyll b (1.66 times) and total carotenoids (1.9 times) increased. Comparing the values obtained for Pb I it was found an accumulation of the amount of chlorophyll a (1.01 times) and chlorophyll b (1.26 times) and an insignificant decrease in the amount of total carotenoids (0.02 times).

Analyzing the results in Figure 1, it was found that the amount of pigments in plants grown in soil containing Cd II, Ni II and Pb II at a maximum allowable concentration increased compared to the control plant. The highest amount of pigments was obtained for plants grown in the presence of Pb II. Thus, in chlorophyll an increase of 1.88 times compared to control, chlorophyll b 1.56 times and total carotenoids 2.15 times. In the case of Cu II, the amount of chlorophyll b increased 1.05 times compared to the control plant; over time the total carotenoids decreased 0.12 times. The decrease in chlorophyll a was insignificant compared to the control (0.02 times). The amount of pigments in plants grown in soil with Zn II and Mn II decreased, except for chlorophyll b in plants grown with Mn II, which increased 1.28 times compared to the control.

Comparing the results obtained for plants grown in soil with heavy metals at a concentration higher than the maximum allowed limit, it was found that in Cd III and Ni III the amount of pigments increased compared to the control. In plants grown in the presence of Cu III, only the amount of chlorophyll b (1.03 times) was higher than the control; chlorophyll a and total carotenoids were lower. By comparing the values obtained for Zn III, an accumulation was found in the case of chlorophyll b (1.16 times) and a decrease in the amount of total carotenoids (0.1 times). The decrease in the amount of chlorophyll a in plants grown in the presence of Zn III compared to the control plant was insignificant (0.05 times). The amount of pigments in plants grown in the presence of Mn III decreased compared to the control plant by 0.38 times in the case of chlorophyll a, 0.34 times in the case of chlorophyll b and 0.37 times in the case of total carotenoids. In the case of plants grown in the presence of Pb III, the amount of chlorophyll a and total carotenoids increased compared to the control, while the amount of chlorophyll b decreased.

#### 2.1.2. Determination of Total Phenolic Content

**Figure 2:**
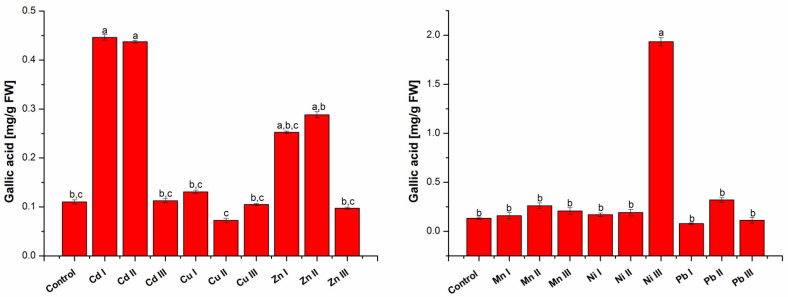
Total polyphenols quantification in lettuce extracts grown in the presence of heavy metals. Each data point is the mean ± the standard error of the mean of three independent replicates experiments; different letters mean significant differences between the treatment and the control plants, determined by Tuckey’s test (*p* < 0.05).

The amount of total polyphenolic compounds presented in Figure 2 was expressed as mg gallic acid/g fresh weight (FW), using the linear equation of the standard calibration curve: y = 0.5865x + 0.0059 (R^2^ = 0.9991).

An increase in the total amount of polyphenols for plants grown in the presence of Cd I, Cu I, Zn I and Ni I can be observed. The highest increase in the total amount of polyphenols was obtained for Cd I (4.05 times) and Zn I (2.29 times). In the case of Mn I, the amount of total polyphenols increased by 1.2 times compared to the control, while in plants grown in soil with Pb I content, the amount of total polyphenols decreased by 0.41 times.

The amount of total polyphenols in plants grown in soil containing Cd II, Ni II and Pb II at a maximum permissible concentration increased compared to the control plant. The highest increase was obtained for Cd II (3.97 times). In the case of plants grown in the presence of Cu II, the amount of total polyphenols decreased compared to the control plant by 0.34 times, and in the case of plants grown in the presence of Zn II and Mn II the amount of total polyphenols increased 2.62 times, respectively, 1.96 times compared to the control.

Comparing the results obtained for plants grown in soil with heavy metals at a concentration higher than the maximum allowed limit, it was found that the amount of total polyphenols in plants grown in the presence of Cd III and Ni III increased compared to control. A significant increase compared to the control was obtained for the amount of total polyphenols (14.51 times) in plants grown in the presence of Ni III. In plants grown in the presence of Cu III, Zn III and Pb III, the amount of total polyphenols decreased, while the amount of total polyphenols in plants grown in the presence of Mn III increased by 1.56 compared to the control.

#### 2.1.3. Establishing Antioxidant Capacity

**Figure 3:**
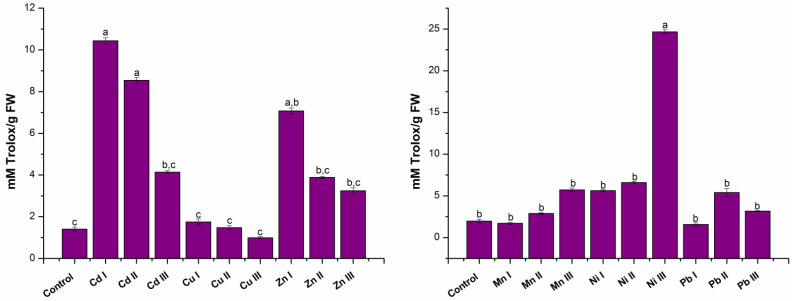
Antioxidant activity of the lettuce extracts. Each data point is the mean ± the standard error of the mean of three independent replicates experiments; different letters mean significant differences between the treatment and the control plants, determined by Tuckey’s test (*p* < 0.05).

The obtained results for antioxidant capacity are presented in Figure 3 and was expressed in mM Trolox equivalents (mM Trolox/g sample), using the linear equation of the standard calibration curve: y = 0.1755x + 0.0198 (R^2^ = 0.9924).

It can be seen that an increase in the antioxidant activity for plants grown in the presence of Cd I, Cu I, Zn I and Ni I increased compared to the control plant; the highest increase was obtained for Cd I 7.43 times and Zn I of 5.04 times. In plants grown in the presence of Mn I and Pb I, the antioxidant activity decreased 0.13 times and 0.2 times compared to the control.

Analyzing the results from plants grown in soil containing Cd II, Ni II and Pb II at a maximum allowable concentration, it was found that the antioxidant activity increased compared to the control plant, the highest increase was obtained for Cd II 6.08 times. In the case of plants grown in the presence of Cu II, Zn II and Mn II, the antioxidant activity increased 1.05 times, 2.76 times, respectively, 1.28 times compared to the control plant.

In the case of plants grown in heavy metal soils at a concentration higher than the maximum allowed limit, it was found that for five of them, the antioxidant activity increased compared to the control. A significant increase compared to the control was obtained for plants grown in the presence of Ni III (12.49 times). In the case of the other plants, an increase in antioxidant activity was observed for Zn III (2.31 times), Mn III (2.9 times), Cd III (2.95 times) and Pb III (1.61 times) compared to the control. Only for plants grown in the presence of Cu III, was there a decrease in antioxidant activity, 0.29 times compared to the control plant.

#### 2.1.4. Elemental Content Determination

Unfortunately, not all samples could be subjected to both long and short irradiation due to the lack of a sufficient amount of the material to be investigated. When this was the case, it was opted for the long irradiation that allows obtaining information about a wider spectrum of elements. A number of 20 elements (Na, Mg, Al, S, Cl, K, Ca, Mn, Fe, Co, Zn, As, Br, Rb, Sr, Sb, Cs, Ba, Sm, Th) were determined in the lettuce leaves (LL) and 16 (Na, K, Fe, Co, Zn, As, Br, Rb, Sr, Sb, Cs, Ba, La, Sm, Th, U) in the root (LR).

As previously mentioned, due to the lack of experimental material, we did not obtain experimental data regarding the influence of all Mn experimental concentrations on the content of Mg, Al, S, Cl, Ca and Mn in leaves. For the same reason, the data regarding the content of the same elements under the influence of Pb (15 and 30 mg kg^−1^), Zn (300 mg kg^−1^) and Ni (75 mg kg^−1^) are missing. 

The literature is quite poor in data regarding the multi-elemental content of salad. Most available studies refer to a very limited number of elements [54,55,56]. The most studied topic is that of the influence of fertilizers and wastewaters to lettuce. In this context, of immediate interest for agriculture is, of course, the green mass of the plant and not the root. That is why it is difficult to extensively compare our data with those obtained by other researchers.

However, Table 1 and Table 2 show that the control values for Na, Mg, Cl, K, Rb, Sb, Cs and Th correlate well at the magnitude unit level with those reported, while lettuce was grown in a soil treated with various phosphate concentration in order to evaluate the efficiency of phosphorus in reducing the availability of different elements to plant [57].

The data for Mn, Fe and Zn reported by Armelin et al. [57] are about a thousand times lower than our data, and those reported in other studies. Our control values for Ca, Co and Br are lower than that of the previously mentioned study. The differences can be attributed to the fact that in our study we use uncontaminated substrate in contrast to the contaminated soil used by the Brazilian researchers, that present values of As, Cd and Cr exceeding the prevention values of 1.5, 33 and 5.3 times, respectively [57]. 

Comparing the experimental data with those in the literature is difficult and questionably relevant, not only due to the fact that few studies are multi-elemental, but also because the elemental content of the lettuce leaves, as shown by Pacheco et al. and Freitas et al., is strongly dependent on the sampling of the leaves. The content of the outer leaves differs significantly from the content of the inner ones. At the same time, it also depends on where the lettuce is grown [58]. 

The content of Na, Mg and Cl is relatively similar to that of lettuce leaves available on the market in Bangkok, Thailand [59], and the central region of Portugal. The content of K, Ca, Fe and Sb is closer to that reported for the central region of Portugal [60] than for Evora and Coimbra [58], while Mn, Co, Zn, As and Br present a higher content in our study than that reported in Portugal. Our data on Al, Fe and Co are much less than those reported from Thailand [59].

The available data regarding the elemental content in lettuce root are even more limited than those regarding the leaves. Our data on element content in lettuce root samples under treatment with different HMs are reported in Table 3. The Zn content in the roots of our samples is comparable to that in those grown in soil contaminated with heavy metals [61], while the Fe content is much higher in our study than in lettuce grown near a highway in Nigeria [62].

#### 2.1.5. Variation of the Elemental Content in Plant Compared to Control

A two-tail *t*-test was used to identify how statistically significant is the difference between the levels of the elemental content in the lettuce leaves and roots and comparison with the control samples. The results are presented in Table 4 and Table 5 for leaves and root, respectively.

The use of Pb induces a more or less significant decrease of the majority of determined elements in leaves (Table 4). Only the Co and As content increased significantly compared to the control at Pb concentrations below 50 mg kg^−1^. In contrast to these, the Br content increases with the application of Pb in higher concentrations. The modification of the soil with Mn also leads to a decrease in concentration for most elements, while the Co content increases significantly. Among decreasing elements, Sb, Sm and Th are significantly affected for all Mn concentrations. The Fe and Zn content scarcely decrease at low Mn concentration but at higher doses, the recession begins evident. In the presence of a low Cu concentration, elements such as Fe, Co, Cs and Ba show a significant increase followed by a more or less significant decrease with the increase in Cu concentration. At the same time, the content of Mn, As and Br increased with the application of Cu. The content of Na, Mg, Ca increases when a high content of Cu is applied. The use of Zn leads to an increase in the content of Zn and Na and a decrease in that of Fe, Sm and Th regardless of the concentration used. The increase in the concentration of Cd in the soil compared to the control leads to a more or less significant increase in the content of Mg, Ca, Mn and Br, but a decrease in the content of Al, Sb, Sm and Th. In the presence of Ni, the content of elements such as Fe, Zn, Sm, Th decreases. The only element showing an increase in concentration for all Ni levels is Br, while Mg, Al, Ca, Mn and Co increases at low Ni concentration and decrease or undergo no significant change at higher Ni levels. It is important to mention that the level of Rb in leaves is not affected by the application of heavy metals which are the subject of the present study. K and S content (with a limited amount of data) is also slightly affected.

At the root level, the presence of heavy metals in the soil clearly induces a significant growth for most of elements (Table 5). The only elements that show significant decreases in content are K and As in the presence of Pb, Mn, Cu and Zn. Rb seems to be not affected by soil amending, except when 30 mg kg^−1^ of Pb is applied to soil.

#### 2.1.6. Elemental Content in Plant Parts versus Content of Applied HMs

**Figure 4:**
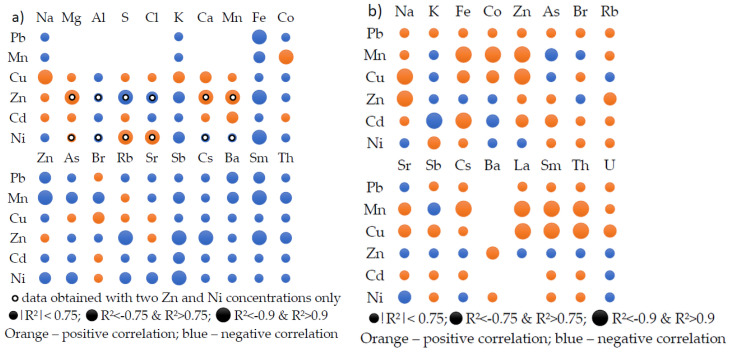
The correlation between the elemental content in the lettuce leaves (**a**) and root (**b**) and the concentration of heavy metals applied to the soil.

The change in the elemental content of the leaf and root samples with the increase in the content of heavy metals applied to the soil is illustrated in Figure 4a,b, respectively. The increase in the Pb concentration in the soil is strongly correlated with the decrease in the Fe content (R^2^ < −0.9), Zn, Ba, Sm (−0.9 < R^2^ < −0.75) in leaves. The root response to increasing Pb concentration is a weakly positive correlation of all elements except Sr.

Cu is the only element whose concentration increase is strongly positively correlated with the content of Na (R^2^ > 0.9), K, Ca and Br (R^2^ > 0.75) in leaves. The behavior of the other elements is weakly correlated with the increase in Cu concentration. Increasing the content of Cu in the soil induces a similar reaction in the root for Na, Zn, La, Sm, Th (R^2^ > 0.9) and Fe, Co, Sb (R^2^ > 0.75). The use of an increasing concentration of Mn only induces an increase in the content of Co in leaves, while the content of the other elements, if it varies, decreases. On the other hand, in the root, the elemental content is strongly correlated for Fe, Co, Zn, Cs, La, Sm. Th.

The increase in Zn concentration appears to be strongly correlated with Mg, Ca, Mn content and uncorrelated with S, but the conclusions are based on incomplete data and therefore cannot be considered definitive; likewise for the influence of Ni on the content of S and Cl. It is worth noting that the content of elements such as S, K, Ca and Fe in leaves is strongly anticorrelated with the increase in Zn content in soil. The increase in the Zn content in the soil goes along with the increase in the content of Na (R^2^ > 0.9), Rb and Ba (R^2^ > 0.75) in root. The variation of Fe and Sb to a greater extent, and K, Zn, As, Rb and Sr more moderately are also contradictory to the variation of soil Ni content. The influence of Ni to the root is moderated: R^2^ > 0.75 for K and R^2^ < −0.75 for Sr.

The change of elemental content in leaves under the influence of Cd is weakly correlated with this. Only Mn on the one hand and Sb on the other hand show correlations of R^2^ > 0.75 and R^2^ < −0.75 respectively. The influence of Cd on elemental content in the root is reflected by a strong positive correlation with Fe content, a moderate one with Zn and As and a negative correlation with K and Co.

We note that, despite the fact that the differences between the content of K and Rb in the experimental and control samples do not seem to be significant (Table 4), when we operate with their average values, the values correlate quite a lot with the variation of the heavy metal concentration added to the soil.

### 2.2. Correlation between Elemental Content and Bioactive Compounds 

**Figure 5:**
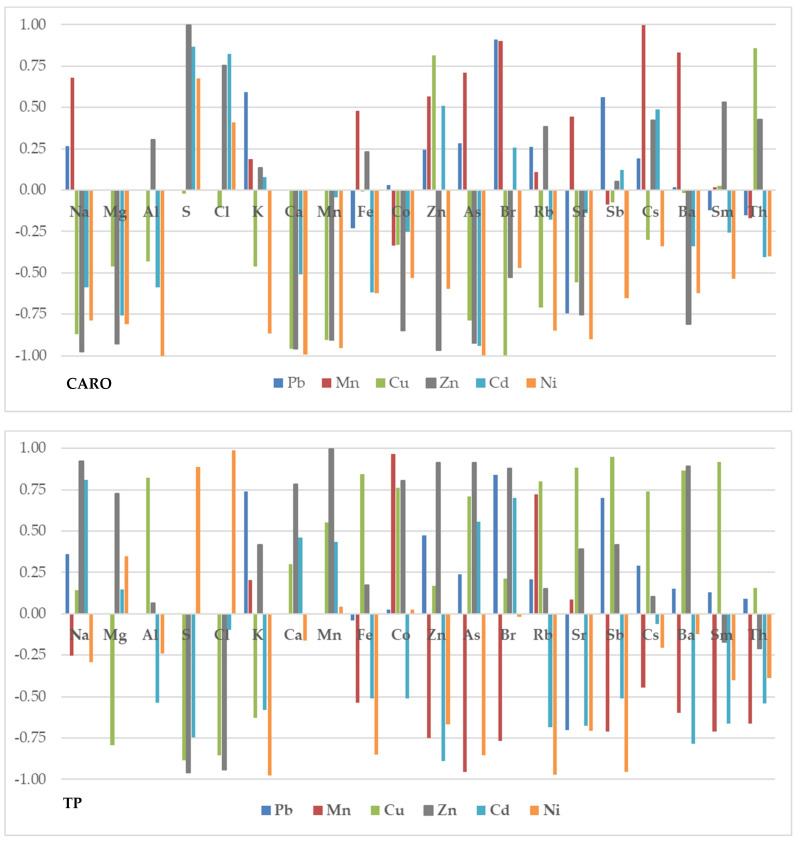

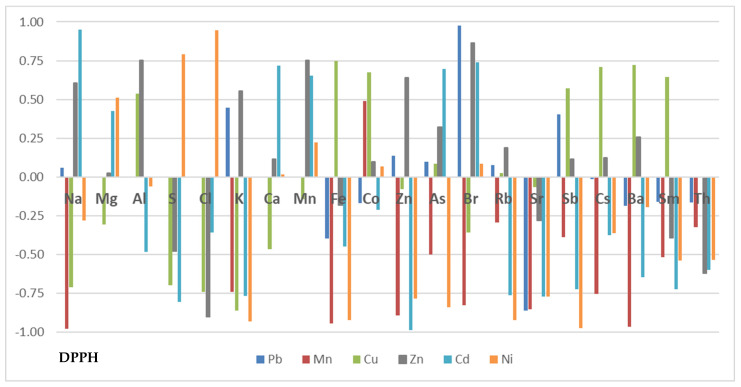
The correlation of the total content of carotenoids (CARO) and polyphenols (TP), as well as the antioxidant activity (DPPH) with the elemental content of lettuce leaves treated with different types of HM.

As Figure 5 shows, for all treatments, in the case of most of the elements, the variation of the elemental content is in dissonance with the variation of the total content of carotenoids and antioxidant activity.

For all determined elements except S and Cl, the use of Ni induces a divergent variation of the elemental content and of carotenoids with correlation coefficients R^2^ < −0.75 for Na, Mg, Al, K, Ca, As, Rb and Sr. A phenomenon similar is induced by the use of Zn and Cd. When adding Cu to the soil, the total content of carotenoids correlates strongly with the content of Zn and Th in the leaves. When applying Pb and Mn, we find that the variation of the carotenoid content is predominantly positively correlated with the elemental content, but it should be remembered that in the case of these two experimental lines no data were obtained for Mg, Al, S, Cl, Ca and Mn. None of the investigated elements shows a significant positive or negative correlation for all the experimental lines. Among the elements determined only for four of the 6 experimental lines, Ca, Mn and Mg show negative correlations for all lines, but not always lower than −0.75.

The correlation between total polyphenolic and elemental content has significant values only for four elements when using Cd (Na: R^2^ > 0.75; S, Zn, Ba: R^2^ < −0.75). When Ni was applied to the soil, the correlation coefficient for K, Fe, As, Rb and Zn is significantly negative and for S and Cl significantly positive. 

The antioxidant activity is weakly correlated with the elemental content then for the experimental line based on Cu, only Cl and K showing significantly negative coefficients and Fe significantly positive. When using Mn, no element was positively correlated with antioxidant activity, instead most elements (Na, K, Fe, Zn, Br, Sr, Cs, Ba) have significantly negative correlation coefficients. We also observe a large number of negative correlations in the case of the experimental line with Ni, but S and Cl are positively correlated with the antioxidant activity.

### 2.3. Translocation Factor

The capacity of the plants to accumulate metals in different parts varies with the plant species and metal type, and can be measured by translocation factor introduced by Yoon et al. in 2006 [6], which related to the ratio of element content in aerial and underground parts of the plant.

In our experiment, the translocation factor was calculated for 14 of 20 determined elements, those which were detected both for leaves and root (Table 6).

Values were ranged from 0.01 for Sb (Pb III) to 4.75 for the Sm control value. As was previously mentioned, the literature is very poor with regard to data on the multielement content of lettuce. Based on the values reported for lettuce grown in soil contaminated with heavy metals [61,63,64] we calculated the translocation factor for Fe and Zn. The obtained values are closed to our values.

For the elements as Fe, Zn, Cs, Ba, Sm, Th the heavy metal addition to soil induced a suppression of the transport process regardless the metal used. The use of Cu leads to a slight increase of translocation factor for Na, K, As, Br, Rb for all concentrations used compared to the control and for Co and Sr at the smallest dose used.

The experimental line with Mn is characterized by an increase of K and As transport from root to leaves at all doses, and Na, Br, Rb when 675 and 1350 mg kg^−1^ were used. The transport of Sr was stimulated only when the lowest concentration was applied.

The use of Cd stimulated the Co and Br transport, but suppressed the transport of other elements regardless the concentration used. 

The delivery of K, As, Br, Rb and Sr was triggered by the lowest dose of Pb, but the highest dose also stimulated the transport of K, Br and Sr. 

It is obvious that the impact of Ni on the transfer factor is minimal compared to the other heavy metals and is difficult to describe in the absence of any evident patterns.

The mobility of K, Br and As are stimulated by the application of most of the experimental doses. In all, 13 of the 18 doses used stimulate the transport of Br, 12 and 11, the transport of K and As, respectively. Of all the heavy metals used, Cu stimulates the transport of the largest number of elements and this happens at all three applied doses.

### 2.4. Cluster Analysis

Cluster analysis was applied to experimental data to give a better insight into the uptake of elements by plants and to assess the contribution of specific factors that may have an effect on plant behavior. 

**Figure 6:**
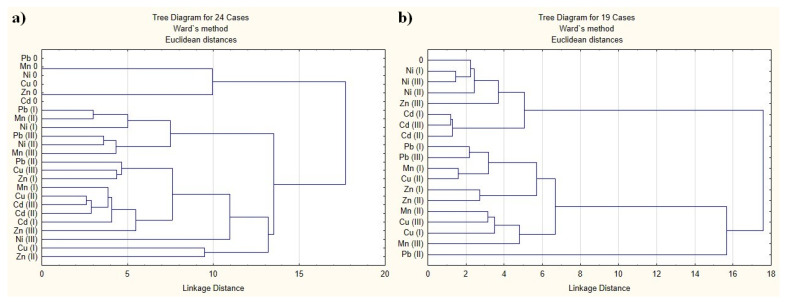
Tree diagram for lettuce leaves based on both elemental and bioactive compounds content (**a**) and for lettuce root based on elemental content (**b**).

For leaves, the analysis was limited to 14 elements which were detected in all investigated samples. The tree diagram (Figure 6a) shows that the control samples are well distinguished from the samples obtained in the presence of heavy metals, the latter being divided into two clusters. The first cluster, less numerous, joins on the one hand the samples subjected to the minimum dose of Pb and Ni, and the intermediate dose of Mn and on the other, the samples obtained at maximum doses of Pb and Mn and the intermediate dose of Ni. The second cluster is more numerous and is also divided into several subclusters, each of them heterogeneous both in terms of the dose applied and the type of heavy metal. In this way, we cannot say that the dose applied has a more important role in the grouping of samples than the type of metal applied, or vice versa.

The cluster analysis for the root samples was performed based on 14 elements that were detected in all samples. The tree diagram (Figure 6b) indicates the formation of two clusters. The first cluster includes two groups of samples, one of them composed of the control sample, all the samples grown in the presence of Ni, and the highest dose of Zn, and the second of all the samples grown in the presence of Cd. The second cluster is much more heterogeneous and includes, on the one hand, the sample grown at the average dose of Pb and on the other hand, all the other samples. We note that the root samples, unlike the leaf samples, are partially grouped according to the type of metal applied, both the samples with Ni and those with Cd being close to the control sample. We can also say that the impact that the minimum and maximum dose of three of the applied elements (Ni, Cd and Pb) have on the roots is quite similar, a fact shown by the minimum distance between them. We cannot say the same about the other three elements.

## 3. Materials and Methods

### 3.1. Chemicals and Materials

For bioactive compounds extraction and analysis ethanol and acetone were purchased from Chimopar, Bucharest, Romania, Folin-Ciocalteu reagent, gallic acid, anhydrous carbonate 2,2′-Diphenyl-picrylhydrazyl (DPPH) and 6-hydroxy-2,5,7,8-tetramethylchroman -2 carboxylic acid (Trolox) were employed from Sigma-Aldrich, Darmstadt, Germany. All chemicals used in the experiments were of analytical grade and the ultrapure water was produced with a Direct-Q^®^ 3 UV Water Purification System, Merck (Darmstadt, Germany).

### 3.2. Plant Growth Conditions

Lettuce seeds (10 grains) were sown at a depth of 1 cm in plastic pots (0.81 L, 13.5 cm in diameter) containing 636 g of garden substrate with active humus and fertilizer for 6 weeks (Agro, 50 L). The physicochemical characteristics of the soil used were pH = 5.5 ± 0.5, N—at least 0.1 m/m%, P_2_O_5_—at least 0.01 m/m%, and K_2_O—at least 0.03 m/m%. The heavy metal salts selected in this study were: copper(II) chloride dihydrate (CuCl_2_·2H_2_O), cadmium acetate dihydrate (Cd(CH_3_COO)_2_·2H_2_O), zinc acetate dihydrate (Zn(CH_3_COO)_2_·2H_2_O), manganese(II) chloride tetrahydrate (MnCl_2_·4H_2_O), nickel chloride (NiCl_2_) and lead(II) sulfate (PbSO_4_). The concentrations of the heavy metal salts were: one under accepted limit (I), one-maximum accepted limit (II) and one above maximum accepted limit (III). These were selected as follows Cu (15, 30, 100 mg kg^−1^); Cd (0.75, 1.5, 3 mg kg^−1^); Zn (75, 150, 300 mg kg^−1^); Mn (675, 1350, 1500 mg kg^−1^); Ni (15, 30, 75 mg kg^−1^) and Pb (15, 30, 50 mg kg^−1^). These salts were dissolved each in 200 mL of ultrapure water, with which the pots were watered. Plant watering was carried out every 4 days with 100 mL of ultrapure water. 

All plants, including control (grown in the absence of heavy metal salts), were grown in a Memmert (ICH260L) climate chamber under controlled light conditions (for 12 h from 24 h), 60% humidity and a day/night temperature cycle of 20/10 °C. Three replicates of each plant were grown. Plant samples were taken 6 weeks after sowing.

### 3.3. Plants and Soil Analysis after Harvesting

#### 3.3.1. Determination of Chlorophylls and Total Carotenoids Content

0.5 g of fresh lettuce leaves are ground with liquid nitrogen in the presence of CaCO_3_ (0.1 g). 10 mL of acetone is added over the thus crushed plant and the grinding is continued for 2 min, after which the clear solution is decanted, and the rest is centrifuged for 10 min at 7000 rpm. Over the remaining plant after decantation, it is adding another 10 mL of acetone and agitated on the shaker at 450 rpm for 30 min, then centrifuge and separates the supernatant. The operation is repeated with another 5 mL of acetone, at the end the plant being discolored. The solutions from the three extraction stages come together in a single bottle. The pigment extraction was performed in triplicate.

The quantitative analysis of chlorophyll a, chlorophyll b and total carotenoids from the obtained extracts was done by UV-VIS spectroscopy using a T80 UV-VIS Spectrophotometer (PG Instruments Limited). In this regard, the absorption spectra of the extracts in the wavelength range 400–750 nm. To determine the pigments concentrations, the following calculation formulas were used [65]:

*c_a_* (mg mL^−1^) = 11.24 × *A*_661.6_ − 2.04 × *A*_644.8_

*c_b_* (mg mL^−1^) = 20.13 × *A*_644.8_ − 4.19 × *A*_661.6_

*c*_(x+c)_ (mg mL^−1^) = (1000 × *A*_470_ − 1.90 × *c_a_* − 63.14 × *c_b_*)/214

where *c_a_* is the concentration of chlorophyll a, *c_b_* is the concentration of chlorophyll b and *c*_(x+c)_ is the concentration of total carotenoids.

#### 3.3.2. Total Polyphenols Evaluation

Fresh lettuce leaves (1 g) were ground in the presence of liquid nitrogen in solvent (15 mL) for 3 min after which the mixture was subjected to ultrasonic-assisted extraction using an Elma Transsonic T ultrasonic bath for 30 min at room temperature. The extraction solvent was 60% ethanol. After extraction, the mixture was centrifuged at 7000 rpm for 10 min and the supernatant was decanted and stored in the refrigerator at 4 °C until analysis. All extracts were obtained in triplicate.

The content of total polyphenols was determined by the Folin-Ciocalteu method [66]. Thus, 1 mL of extract and 0.5 mL of Folin-Ciocalteu reagent was added to a 10 mL volumetric flask containing 5 mL of double distilled water. The content of the flask was mixed and after 3 min of standing, 1.5 mL of Na_2_CO_3_ (5 g L^−1^) was added and the volume of the flask was adjusted with double distilled water. The samples were placed in a water bath at 50 °C, where they were kept for 16 min, after which were removed and allowed to cool to room temperature. The absorbances of the samples were read in relation to the double distilled water at 765 nm. 

To determine the total amount of polyphenols, a calibration curve was drawn using as standard a gallic acid solutions in the range of 0.001–0.800 mg mL^−1^. Gallic acid concentrations were obtained by successive dilutions with double distilled water starting from a stock solution with a concentration of 1 mg mL^−1^.

#### 3.3.3. Antioxidant Capacity Determination by DPPH Method

A slightly modified procedure of Brand-Williams et al. [67] was used for the antioxidant activity determination. Thus, 0.01 mL of extract was added to 3.9 mL of DPPH-2,2 diphenyl-picryl-hydrazyl radical solution (0.0025 g/100 mL methanol). The mixture was left in the dark for 10 min, after that the absorbance of the mixture was measured at 515 nm comparative to a mixture obtained from 0.01 mL extract added to 3.9 mL methanol. The antioxidant activity was determined using a calibration curve drawn for different concentrations of Trolox (0–400 µM).

### 3.4. Multielemental Investigation of Lettuce and Soil Substrate by NAA

To determine the elemental content of the lettuce biomass and soil substrate, neutron activation analysis (NAA) at the pulsed fast reactor IBR-2 (FLNP JINR, Dubna) was used. A total number of 55 plant samples and 28 soil samples were analyzed. To determine short-lived isotopes, biological (about 0.3 g) and substrate samples (about 0.1 g) were irradiated for 3 min and 1 min, respectively, under a thermal neutron fluency rate of approximately 1.6 × 10^13^ n cm^−2^ s^−1^. Both types of samples were measured for 15 min. In the case of long-lived isotopes substrate (about 0.1 g) samples were irradiated for 3 days under a resonance neutron fluency rate of approximately 3.31 × 10^12^ n cm^−2^ s^−1^, repacked and measured using high purity germanium detectors twice (after 4–5 days and 20–23 days of decay).

### 3.5. Statistical Analysis

The obtained results are presented as the mean of three measurements ± SD (standard deviation). In order to evaluate the statistically significant differences between groups (*p* < 0.05), one-way analysis of variance (ANOVA) followed by Tukey’s test performed with Minitab 17 (Minitab Ltd., Coventry, UK) were used. 

## 4. Conclusions

The paper evaluated the variation of the content of biologically active compounds, elemental content and the antioxidant activity in the plants grown in contaminated soil with different concentration of Cu, Cd, Mn, Ni, Zn and Pb, compared to the control plants.

The amount of chlorophyll a, chlorophyll b, total carotenoids and polyphenols depends on metal from soil and their concentration. 

The antioxidant activity of plants grown in the presence of heavy metals was higher than in control plants, except for plants grown in the presence of Cu at a concentration above the maximum accepted limit, Mn and Pb at a concentration under accepted limit. For these plants, the antioxidant activity was lower than in the control plants.

The use of Pb and Mn induces a decrease in the concentration of most elements compared to the control and this decrease is usually strongly correlated with the increase in the heavy metal concentration in the soil. For low doses of Cu, Cd and Ni we notice an increased number of elements with a significant increase in concentration compared to the impact of higher doses. The dose of Zn with the most stimulating effect seems to be the average one. 

At the root level, the presence of heavy metals in the soil clearly induces a significant increase for most elements. The relationship between the elemental content in the leaves and bioactive compounds content is dependent on the experimental line, none of the investigated elements showing us a significant positive or negative correlation between the elemental content and total carotenoids for all the experimental lines. For elements such as Fe, Zn, Cs, Ba, Sm and Th, the addition of heavy metals to the soil induced a suppression of the root-to-leaf transport process, regardless of the metal used. Of all the heavy metals applied, Cu stimulates the transport of the largest number of elements and this happens at all three applied doses. The only characteristic that categorically divides the lettuce leaves samples into categories is the amendment or not of the soil with the help of heavy metals. The cluster analysis of the roots, based on the elemental content, highlights the fact that the samples grown in the presence of Ni and Cd group together, respectively. 

In conclusion, the plants were influenced by the presence in soil of selected metals and their composition depends on metal quantity.

## Figures and Tables

**Table 1:** Elemental content in lettuce leaves samples (LL) under treatment with different HMs.

	Pb	Mn	Cu	Zn	Cd	Ni	Control
Na *	3.2–4.5	3.2–4.4	3.8–6.0	4.4–7.1	4.0–4.6	3.5–4.9	3.8–4.3
Mg *	2.7–3.1 ^a^	-	2.7–3.9	2.6–3.9 ^a^	2.5–3.7	2.7–4.3 ^a^	2.5–3.0
Al **	70.4–79.6 ^a^	-	133–660	177–220 ^a^	96–181	132–293 ^a^	183–203
S ^a^ **	7.2–11.8 ^a^	-	4.8–15.6	5.3–10.1 ^a^	5.0–12.6	7.7–15.2 ^a^	7.2–12.7
Cl *	30.6–35.0 ^a^	-	22.7–42.9	22.1–26.8 ^a^	25.3–36.1	32.7–40.4 ^a^	30.3–34.7
K *	93.0–129	91.2–130	98.3–136	87.6–136	96.5–119	82.8–132	101–125
Ca *	9.0–11.7 ^a^	-	10.5–17.7	11.6–25.2 ^a^	11.4–17.2	8.0–17.7 ^a^	9.7–12.7
Mn **	92–106 ^a^	-	146–409	1256–1648 ^a^	229–292	84–389 ^a^	112–130
Fe **	144–308	155–295	181–527	141–279	139–304	174–279	278–334
Co ***	58–248	17–306	93–360	74–242	95–146	94–201	102–134
Zn **	111–286	197–302	102–137	729–1757	86–242	100–209	289–329
As **	0.49–0.81	0.48–0.61	0.54–0.96	0.50–0.97	0.54–0.82	0.47–0.63	0.54–0.59
Br **	17.6–49.9	13.7–28.9	33.0–61.8	17.6–49.9	34.8–53.1	28.0–53.1	21.2–25
Rb **	40.1–65.2	40.0–65.2	40.1–61.7	35.9–57.0	38.5–54.7	32.6–60.5	41.0–57.0
Sr **	20.9–42.0	22.2–38.6	24.6–40.7	24.8–47.3	24.9–32.4	21.0–35.3	28.7–35.3
Sb ***	11.5–68.9	25.7–35.7	21.5–76.5	12–53.2	20–33.8	26.7–54.2	39.4–56.6
Cs ***	46.4–82.6	58.5–83.5	61.1–133	48.3–76.8	50.7–74.0	53.2–71.0	62.6–75.4
Ba **	7.3–15.7	5.3–16.4	6.8–22.0	7.8–21.4	7.5–14.9	6.4–16.8	10.2–15.0
Sm ***	8.1–24.7	6.4–16.1	14.3–70.1	6.4–26.1	12.0–31.1	11–33.5	28.9–47.1
Th ***	15.9–36.9	15.0–28.5	15.0–25.0	7.5–37.5	7.5–41.6	24.1–43.4	58–68

^a^ partial data; * g kg^−1^; ** mg kg^−1^; *** µg kg^−1^.

**Table 2:** Literature data on elemental content of lettuce leaves.

	[45]		[47]				[48]	[49]			
	CLL	LL	ILL (Evora)	OLL (Evora)	ILL (Coimbra)	OLL (Coimbra)	LL	OLL	OLL	ILL	ILL
Na *	4.2	6–10.3	0.14 ± 0.03	0.34 ± 0.07	0.17 ± 0.14	0.31 ± 0.11	2.9 ± 7	1.93 ± 0.01	2.4 ± 0.8	0.74 ± 0.04	1.3 ± 1.1
Mg *	5.2	3.6–6.8					3.6 ± 0.7	3.2 ± 0.014		2.04 ± 0.01	
Al **							3524 ± 318				
S *											
Cl *	22.8	8.5–15.1					17.9 ± 0.4				
K *	96.1	52.7–74.9	6.3 ± 1.4	8.3 ± 1.9	7.2 ± 0.8	10.7 ± 1.7		84.8 ± 1.7	82 ± 13	54.3 ± 0.1	55.1 ± 6
Ca *	30.3	14.9–22.9	0.51 ± 0.08	1.17 ± 0.18	0.54 ± 0.05	1.29 ± 0.06	13.3 ± 0.6	12.9 ± 0.15	9.92 ± 0.45	5.0 ± 0.28	4.18 ± 0.04
Mn **	0.042	0.013–0.038					68.81 ± 0.22	157.6 ± 3.1		38.2 ± 1.2	
Fe **	0.42	0.19–0.97	16.1 ± 3.0	44 ± 13	16.9 ± 4.6	53.2 ± 3.6	1506 ± 54.3 ^NAA^ 530.4 ^AAS^	343 ± 24	409 ± 28	102 ± 10	130 ± 35
Co ***	302	76–1026	7.9 ± 1.7	19.3 ± 5.6	8.4 ± 1.7	30 ± 10	1010 ± 160 ^NAA^ 7200 ^AAS^	0.13 ± 0.05	0.23 ± 0.08	0.067 ± 0.02	
Zn **	0.84	0.35–1.2	4.99 ± 0.58	3.55 ± 0.6	7.24 ± 0.5	7.14 ± 0.33		45.7 ± 1	54.9 ± 2.5	56.2 ± 3.9	55.7 ± 3.8
As ***			13 ± 6.7	43 ± 16	6.5 ± 2.1	24.3 ± 2.1					
Br **	87.5	62–80	1.27 ± 0.23	2.74 ± 0.45	3.41 ± 0.45	8.3 ± 1.8					
Rb **	52.3	59.3–81	1.19 ± 0.1	1.56 ± 0.11	1.2 ± 0.12	1.72 ± 0.18		11.6 ± 0.94	13.2 ± 1.4	9.22 ± 0.34	9.23 ± 0.92
Sr **			2.45 ± 0.39	5.5 ± 0.54	2.00 ± 0.23	6.85 ± 0.59					
Sb ***	85	30.3–79	<8	3.94 ± 0.86	4.5 ± 1.5	11.5 ± 1.8		46.8 ± 7.1	88 ± 14	34.9 ± 7.3	35 ± 12
Cs ***	50	170–2360									
Ba											
Sm											
Th ***	25	24–143									

* g kg^−1^; ** mg kg^−1^; *** µg kg^−1^; CLL—control lettuce leaves; OLL—outer lettuce leaves; ILL—inner lettuce leaves; NAA—Neutron Activation Analysis; AAS—Atomic Absorption Spectrometry.

**Table 3:** Elemental content in lettuce root samples (LR) under treatment with different HMs.

	Pb	Mn	Cu	Zn	Cd	Ni	Control
Na *	5.2–10.9	4.4–6.7	4.7–7.2	5.0–6.9	5.4–7.5	5.1–7.6	4.9–5.6
K *	114–243	92.7–150	78.3–134	112–162	135–173	140–182	142–172
Fe **	1811–7194	1398–3960	1033–2554	394–2494	285–475	317–474	259–351
Co **	1.75–10.0	2.5–6.3	1.03–3.4	0.66–5.7	0.61–1.12	0.69–2.4	1.17–1.53
Zn **	116–485	140–286	121–234	1896–3347	96.6–152	103–312	82.9–97.1
As **	4.0–6.7	2.9–4.0	5.3–7.2	4.0–7.3	11.7–17.0	5.1–9.0	6.1–6.9
Br **	40.3–158	33.9–57.5	51.4–79.1	43.1–80.1	66.9–95.3	75.2–103	49.5–58.5
Rb **	51.8–129	42.6–87.4	39.4–63.6	51.0–77.7	55.2–79.2	56.8–82.6	52.6–73.4
Sr **	27.4–125	30.4–155	32.3–77.0	36.4–75.1	36.7–61.1	36.5–56.4	40.0–54.1
Sb ***	87.7–4510	78.9–2251	67.2–1839	82.7–1819	69.6–157	77.1–209	120–159
Cs ***	216–975	167–686	209–819	133–422	124–201	100–485	98.6–147
Ba **	-	-	-	38.9–67.3	-	23.9–45.6	14.3–23.8
La **	1.23–4.4	0.83–2.3	0.57–2.8	0.54–2.2	-	-	0.62–0.84
Sm ***	234–699	128–279	105–410	24.6–26.9	17.8–46.9	12.5–49.1	5.6–10.4
Th ***	225–1262	203–602	234–766	45.0–423	52.5–123	67.5–152	45.0–75.0
U ***	157–304	94.4–278	85.3–230	67.8–230	67.3–133	75.4–187	121–177

* g kg^−1^; ** mg kg^−1^; *** µg kg^−1^.

**Table 4:** The level of significance of the difference compared to the content of the control sample in lettuce leaves.

	Pb			Mn			Cu			Zn			Cd			Ni		
mg kg^−1^	I	II	III	I	II	III	I	II	III	I	II	III	I	II	III	I	II	III
Na	-	-	*	-	-	*	-	-	***	***	***	**	*	-	-	*	-	-
Mg			-				-	***	**	-	**		**	-	*	***	-	
Al			***				***	***	***	-	-		*	***	***	***	***	
S			-				-	-	-	-	**		-	-	-	-	-	
Cl			-				***	***	**	***	***		**	-	-	-	*	
K	-	-	-	-	-	-	-	-	-	-	-	-	-	-	-	-	-	*
Ca			-				-	-	**	-	***		**	-	*	**	-	
Mn			**				***	***	***	***	***		***	***	***	***	***	
Fe	-	**	***	-	*	***	***	***	***	*	**	***	-	***	*	*	*	***
Co	***	**	***	***	***	***	***	-	-	-	***	*	-	-	-	***	-	-
Zn	***	*	***	-	***	***	***	***	***	***	***	***	***	***	***	***	***	***
As	***	***	-	-	-	-	***	-	***	***	***	-	***	-	-	-	-	**
Br	*	***	***	-	***	***	***	***	***	***	***	*	***	***	***	***	***	***
Rb	-	-	-	-	-	-	-	-	-	-	-	-	-	-	-	-	-	-
Sr	-	**	*	-	-	**	-	-	-	-	*	-	-	-	-	-	*	**
Sb	-	*	***	**	**	**	**	**	-	-	-	***	**	**	***	-	-	*
Cs	-	-	**	-	-	-	***	-	-	-	-	-	-	-	*	-	-	-
Ba	-	-	*	-	-	**	**	*	-	-	*	-	-	-	-	-	*	-
Sm	***	**	***	***	***	***	-	**	-	**	**	***	-	***	*	-	***	**
Th	***	***	***	***	***	***	***	***	***	***	***	***	***	***	***	***	***	***

empty cell—not determined due to lack of data; “-“ no significant difference, “*” α = 0.1; “**” α = 0.5; “***” α = 0.02; red—positive difference, blue—negative difference.

**Table 5:** The level of significance of the difference compared to the content of the control sample in lettuce root.

	Pb			Mn			Cu			Zn			Cd			Ni		
mg kg^−1^	I	II	III	I	II	III	I	II	III	I	II	III	I	II	III	I	II	III
Na	**	***	-	-	-	-	-	-	**	-	-	*	**	-	-	**	-	-
K	*	***	-	**	***	-	***	**	***	*	*	-	-	-	-	-	-	-
Fe	***	***	***	***	***	***	***	***	***	***	***	**	-	-	**	**	-	-
Co	***	***	**	***	***	***	***	-	***	***	***	***	*	***	***	***	***	-
Zn	***	***	***	***	***	***	***	***	***	***	***	***	***	*	***	***	**	***
As	***	-	-	***	***	***	-	**	-	***	***	-	***	***	***	**	**	***
Br	**	***	***	-	***	-	-	***	-	***	-	-	***	***	***	***	***	***
Rb	-	**	-	-	-	-	-	-	-	-	-	-	-	-	-	-	-	-
Sr	-	**	*	-	**	**	-	-	*	-	*	-	-	-	-	-	-	-
Sb	***	***	***	***	***	***	***	***	***	***	***	*	-	-	**	-	**	-
Cs	***	***	***	**	***	***	***	***	***	***	***	-	-	**	-	-	***	-
Ba										***	***	***				*	**	**
La	***	***	***	*	***	***	***	-	***	***	*	-	-	-	-	-	-	-
Sm	***	***	***	***	***	***	***	***	***	***	***	***	***	***	***	***	**	***
Th	***	***	***	***	***	***	***	***	***	***	***	-	***	-	-	***	-	***
U	-	**	-	-	-	*	-	-	**	-	*	**	**	-	*	-	*	-

empty cell—not determined due to lack of data; “-“ no significant difference, “*” α = 0.1; “**” α = 0.5; “***” α = 0.02; red—positive difference, blue—negative difference.

**Table 6:** Translocation factor of lettuce.

	mg kg^−1^	Na	K	Fe	Co	Zn	As	Br	Rb	Sr	Sb	Cs	Ba	Sm	Th
C	0	0.74	0.72	1.00	0.09	3.43	0.09	0.43	0.78	0.68	0.33	0.56	0.66	4.75	1.05
Pb	I	0.64	**0.86**	0.11	0.09	1.45	**0.18**	**0.44**	**0.88**	**0.75**	0.04	0.19		0.08	0.07
	II	0.42	0.53	0.04	0.02	0.60	**0.11**	0.32	0.50	0.24	0.02	0.09		0.03	0.03
	III	0.61	**0.76**	0.08	0.04	0.66	0.09	**0.44**	0.76	**0.78**	0.01	0.20		0.07	0.08
Mn	I	**0.85**	**0.93**	0.17	0.07	1.87	**0.19**	**0.50**	**0.95**	**0.87**	0.04	0.37		0.09	0.08
	II	**0.83**	**1.15**	0.07	0.06	1.26	**0.16**	**0.45**	**1.08**	0.43	0.02	0.12		0.05	0.05
	III	0.56	**0.76**	0.06	0.04	0.79	**0.14**	0.29	0.66	0.21	0.02	0.11		0.03	0.05
Cu	I	**0.87**	**1.20**	0.25	**0.14**	0.85	**0.13**	**0.84**	**1.10**	**0.97**	0.08	0.17		0.34	0.06
	II	**0.78**	**0.98**	0.18	0.09	0.71	**0.10**	**0.49**	**0.89**	0.59	0.04	0.29		0.16	0.08
	III	**0.85**	**1.40**	0.09	0.04	0.58	**0.14**	**0.92**	**0.96**	0.55	0.02	0.15		0.07	0.03
Zn	I	**1.04**	**0.98**	0.11	0.02	0.61	**0.17**	**0.62**	**0.79**	0.54	0.03	0.18	0.26	0.08	0.05
	II	**1.18**	**0.82**	0.20	**0.10**	0.53	**0.19**	**0.58**	0.68	0.64	0.04	0.21	0.37	0.15	0.12
	III	**0.75**	0.66	0.34	**0.11**	0.38	0.08	0.41	0.65	**0.77**	0.16	0.35	0.18	0.29	0.17
Cd	I	0.68	0.68	0.76	**0.12**	0.71	0.05	**0.51**	0.69	0.67	0.20	0.41		0.61	0.35
	II	0.72	0.70	0.44	**0.16**	1.56	0.05	**0.56**	0.69	0.66	0.20	0.37		0.67	0.14
	III	0.73	0.72	0.59	**0.18**	1.60	0.04	**0.52**	0.71	0.53	0.30	0.36		0.92	0.34
Ni	I	0.66	**0.75**	0.61	0.08	0.67	0.08	**0.52**	0.73	0.64	0.23	0.45	0.47	0.84	0.29
	II	0.64	**0.75**	0.65	**0.14**	1.37	**0.10**	0.37	0.75	0.61	**0.51**	0.13	0.22	0.83	0.31
	III	0.70	0.55	0.50	**0.12**	0.86	0.06	0.38	0.56	0.50	0.23	0.53	0.35	0.51	0.25

Bold—values exceeding control. [52] Fe:1.37/0.94; Zn:0.0009/0.0026. [53] Zn: 0.94. [50] Zn: 0.625.

## Data Availability

Not applicable.

## Sample Availability

Not available.

## References

[1] Xie N., Kang C., Ren D., Zhang L. (2022). Assessment of the variation of heavy metal pollutants in soil and crop plants through field and laboratory tests. Sci. Total Environ..

[2] Sanjosé I., Navarro-Roldán F., Infante-Izquierdo M.D., Martínez-Sagarra G., Devesa J.A., Polo A., Ramírez-Acosta S., Sánchez-Gullón E., Jiménez-Nieva F.J., Muñoz-Rodríguez A.F. (2021). Accumulation and effect of heavy metals on the germination and growth of *Salsola vermiculata* L. seedlings. Diversity.

[3] Klink A. (2017). A comparison of trace metal bioaccumulation and distribution in *Typha latifolia* and *Phragmites australis*: Implication for phytoremediation. Environ. Sci. Pollut. Res..

[4] Agrawal S.B., Singh A., Sharma R.K., Agrawal M. (2007). Bioaccumulation of heavy metals in vegetables: A threat to human health. Terr. Aquat. Environ. Toxicol..

[5] Gope M., Masto R.E., George J., Hoque R.R., Balachandran S. (2017). Bioavailability and health risk of some potentially toxic elements (Cd, Cu, Pb and Zn) in street dust of Asansol, India. Ecotoxicol. Environ. Saf..

[6] Yoon J., Cao X., Zhou Q., Ma L.Q. (2006). Accumulation of Pb, Cu, and Zn in native plants growing on a contaminated Florida site. Sci. Total Environ..

[7] Ali W., Mao K., Zhang H., Junaid M., Xu N., Rasool A., Feng X., Yang Z. (2020). Comprehensive review of the basic chemical behaviours, sources, processes, and endpoints of trace element contamination in paddy soil-rice systems in rice-growing countries. J. Hazard. Mater..

[8] Sihlahla M., Mouri H., Nomngongo P.N. (2019). Uptake of trace elements by vegetable plants grown on agricultural soils. J. Afr. Earth Sci..

[9] Cabral-Pinto M.M., Inácio M., Neves O., Almeida A.A., Pinto E., Oliveiros B., Ferreira da Silva E.A. (2020). Human health risk assessment due to agricultural activities and crop consumption in the surroundings of an industrial area. Expos. Health.

[10] Tong S., Yang L., Gong H., Wang L., Li H., Yu J., Li Y., Deji Y., Nima C., Zhao S. (2022). Bioaccumulation characteristics, transfer model of heavy metals in soil-crop system and health assessment in plateau region, China. Ecotoxicol. Environ. Saf..

[11] Kunhikrishnan A., Bolan N.S., Müller K., Laurenson S., Kim W.I. (2012). The influence of wastewater irrigation on the transformation and bioavailability of heavy metal(loid)s in soil. Adv. Agron..

[12] Muhammad M.S. (2013). Human health risk from heavy metal via food crops consumption with wastewater irrigation practices in Pakistan. Chemosphere.

[13] Xi B.D., Yu H., Li Y.P., Dang Q.L., Tan W.B., Wang Y., Cui D.Y. (2020). Insights into the effects of heavy metal pressure driven by long-term treated wastewater irrigation on bacterial communities and nitrogen-transforming genes along vertical soil profiles. J. Hazard. Mater..

[14] Xiao B., Xue P.Y., Wei L., Liu C.C. (2020). Characteristics of Cd, As, and Pb in soil and wheat grains and health risk assessment of grain-Cd/As/Pb on the field scale. Environ. Sci..

[15] Nodelkoska T.V., Doran P.M. (2000). Interactive effects of temperature and metal stress on the growth and some biochemical compounds in wheat seedlings. Environ. Pollut..

[16] Diaconu M., Pavel L.V., Hlihor R.M., Rosca M., Fertu D.I., Lenz M., Corvini P.X., Gavrilescu M. (2020). Characterization of heavy metal toxicity in some plants and microorganisms–A preliminary approach for environmental bioremediation. Nat. Biotechnol..

[17] Rehman A.U., Nazir S., Irshad R., Tahir K., ur Rehman K., Islam R.U., Wahad Z. (2020). Toxicity of heavy metals in plants and animals and their uptake by magnetic iron oxide nanoparticles. J. Mol. Liq..

[18] Clemens S., Ma J.F. (2016). Toxic heavy metal and metalloid accumulation in crop plants and foods. Annu. Rev. Plant Biol..

[19] Seneviratne M., Rajakaruna N., Rizwan N., Madawala H.M.S.P., Ok Y.S., Vithanage M. (2019). Heavy metal-induced oxidative stress on seed germination and seedling development: A critical review. Environ. Geochem. Health.

[20] Jin C., Fan J., Liu R., Sun R. (2015). Single and joint toxicity of sulfamonomethoxine and Cadmium on three agricultural crops. Soil Sediment Contam..

[21] Mirshekali H., Hadi H., Amirnia R., Khodaverdiloo H. (2012). Effect of zinc toxicity on plant productivity, chlorophyll and Zn contents of Sorghum (*Sorghum bicolor*) and common Lambsquarter (*Chenopodium album*). Int. J. Agric. Res..

[22] Tauqeer H.M., Ali S., Rizwan M., Ali Q., Saeed R., Iftikhar U., Ahmad R., Farid M., Abbasi G.H. (2016). Phytoremediation of heavy metals by Alternanthera bettzickiana: Growth and physiological response. Ecotoxicol. Environ. Saf..

[23] Zhao H., Guan J., Liang Q., Zhang X., Hu H., Zhang J. (2021). Effects of cadmium stress on growth and physiological characteristics of sassafras seedlings. Sci. Rep..

[24] Andrade Júnior W.V., Oliveira Neto C.F., Santos Filho B.G., Amarante C.B., Cruz E.D., Okumura R.S., Barbosa A.V.C., Sousa D.J.P., Teixeira J.S.S., Botelho A.S. (2019). Effect of cadmium on young plants of *Virola surinamensis*. AoB Plants.

[25] Grajek H., Rydzyński D., Piotrowicz-Cieślak A., Herman A., Maciejczyk M., Wieczorek Z. (2020). Cadmium ion-chlorophyll interaction–Examination of spectral properties and structure of the cadmium-chlorophyll complex and their relevance to photosynthesis inhibition. Chemosphere.

[26] Mwamba T.M., Ali S., Ali B., Lwalaba J.L., Liu H., Farooq M.A., Shou J., Zhou W. (2016). Interactive effects of cadmium and copper on metal accumulation, oxidative stress, and mineral composition in *Brassica napus*. Int. J. Environ. Sci. Technol..

[27] Laporte D., Rodríguez F., González A., Zúñiga A., Castro-Nallar E., Sáez C.A., Moenne A. (2020). Copper-induced concomitant increases in photosynthesis, respiration, and C, N and S assimilation revealed by transcriptomic analyses in *Ulva compressa* (Chlorophyta). BMC Plant Biol..

[28] Wang Z., Zhang J., Li E., Zhang L., Wang X., Song L. (2017). Combined toxic effects and mechanisms of microsystin-LR and copper on *Vallisneria Natans* (Lour.) Hara seedlings. J. Hazard. Mater..

[29] Yagura R., Imanishi J., Shibata S. (2019). Effects of copper ions on the growth and photosynthetic activity of *Scopelophila cataractae*. Lindbergia.

[30] Dey S., Mazumder P.B., Paul S.B. (2014). Effect of copper on growth and chlorophyll content in tea plants (*Camellia sinensis* (L.) O. Kuntze). Int. J. Res. Appl. Nat. Soc. Sci..

[31] Giannakoula A., Therios I., Chatzissavvidis C. (2021). Effect of lead and copper on photosynthetic apparatus in citrus (*Citrus aurantium* L.) plants. The role of antioxidants in oxidative damage as a response to heavy metal stress. Plants.

[32] Aghajanzadeh T.A., Prajapati D.H., Burow M. (2020). Differential partitioning of thiols and glucosinolates between shoot and root in Chinese cabbage upon excess zinc exposure. J. Plant Physiol..

[33] Paunov M., Koleva L., Vassilev A., Vangronsveld J., Goltsev V. (2018). Effects of different metals on photosynthesis: Cadmium and zinc affect chlorophyll fluorescence in durum wheat. Int. J. Mol. Sci..

[34] Islam F., Yasmeen T., Riaz M., Arif M.S., Ali S., Raza S.H. (2014). Proteus mirabilis alleviates zinc toxicity by preventing oxidative stress in maize (*Zea mays*) plants. Ecotoxicol. Environ. Saf..

[35] Betancourt O., Tapia M., Méndez I. (2015). Decline of general intelligence in children exposed to manganese from mining contamination in Puyango river basin, Southern Ecuador. Ecohealth.

[36] Fernando D.R., Lynch J.P. (2015). Manganese phytotoxicity: New light on an old problem. Ann. Bot..

[37] Sieprawska A., Filek M., Tobiasz A., Walas S., Dudek-Adamska D., Grygo-Szymanko E. (2016). Trace elements’ uptake and antioxidant response to excess of manganese in in vitro cells of sensitive and tolerant wheat. Acta Physiol. Plant..

[38] Yang S.X., Deng H., Li M.S. (2008). Manganese uptake and accumulation in a woody hyperaccumulator, Schima superba. Plant Soil Environ..

[39] Li J., Jia Y., Dong R., Huang R., Liu P., Li X., Wang Z., Liu G., Chen Z. (2019). Advances in the mechanisms of plant tolerance to manganese toxicity. Int. J. Mol. Sci..

[40] He L., Su R., Chen Y., Zeng P., Du L., Cai B., Zhang A., Zhu H. (2022). Integration of manganese accumulation, subcellular distribution, chemical forms, and physiological responses to understand manganese tolerance in *Macleaya cordata*. Environ. Sci. Pollut. Res..

[41] Gill R.A., Kanwar M.K., Rodrigues dos Reis A., Ali B. (2022). Editorial: Heavy metal toxicity in plants: Recent insights on physiological and molecular aspects. Front. Plant Sci..

[42] Gupta V., Jatav P.K., Verma R., Kothari S.L., Kachhwaha S. (2017). Nickel accumulation and its effect on growth, physiological and biochemical parameters in millets and oats. Environ. Sci. Pollut. Res. Int..

[43] Singh G., Agnihotri R.K., Reshma R.S., Ahmad M. (2012). Effect of lead and nickel toxicity on chlorophyll and proline content of Urd (*Vigna mungo* L.) seedlings. Int. J. Plant Physiol. Biochem..

[44] Cenkci S., Cigerci I.H., Yıldız M., Ozay C., Bozdag A., Terzi H. (2010). Lead contamination reduces chlorophyll biosynthesis and genomic template stability in *Brassica rapa* L.. Environ. Exp. Bot..

[45] Afaj A.H., Jassim A.J., Noori M.M., Schuth C. (2016). Effects of lead toxicity on the total chlorophyll content and growth changes of the aquatic plant *Ceratophyllum demersum* L.. Int. J. Environ. Stud..

[46] Sharma P., Dubey R.S. (2005). Lead toxicity in plants. Braz. J. Plant Physiol..

[47] Ahmed A., Tajmir–Riahi H.A. (1993). Interaction of toxic metal ions Cd^2+^, Hg^2+^ and Pb^2+^ with light–harvesting proteins of chloroplast thylakoid membranes. An FTIR spectroscopic study. J. Inorg. Biochem..

[48] Vodnik D., Jentschke G., Fritz E., Gogala N., Godbold D.L. (1999). Root–applied cytokinin reduces lead uptake and affects its distribution in Norway spruce seedlings. Physiol. Plant.

[49] Su R., Ou Q., Wang H., Luo Y., Dai X., Wang Y., Chen Y., Shi L. (2022). Comparison of phytoremediation potential of *Nerium indicum* with inorganic modifier calcium carbonate and organic modifier mushroom residue to lead-zinc tailings. Int. J. Environ. Res. Public Health.

[50] Yu C., Yan C., Liu Y., Liu Y., Jia Y., Lavelle D., An G., Zhang W., Zhang L., Han R. (2020). Upregulation of a KN1 homolog by transposon insertion promotes leafy head development in lettuce. Proc. Natl. Acad. Sci. USA.

[51] Lopez A., Javier G.A., Fenoll J., Hellín P., Flores P. (2014). Chemical composition and antioxidant capacity of lettuce: Comparative study of regular-sized (Romaine) and baby-sized (Little Gem and Mini Romaine) types. J. Food Compos. Anal..

[52] Nicolle C., Carnat A., Fraisse D., Lamaison J.L., Rock E., Michel H., Amouroux P., Remesy C. (2004). Characterisation and variation of antioxidant micronutrients in lettuce (*Lactuca sativa folium*). J. Sci. Food Agric..

[53] Chaney R.L. (2015). How does contamination of rice soils with Cd and Zn cause high incidence of human Cd disease in subsistence rice farmers. Curr. Pollut. Rep..

[54] Achakzai A.K.K., Bazai Z.A., Kayani S.A. (2011). Accumulation of heavy metals by lettuce (*Lactuca sativa* L.) irrigated with different levels of wastewater of Quetta city. Pak. J. Bot..

[55] Ferri R., Donna F., Smith D.R., Guazzetti S., Zacco A., Rizzo L., Bontempi E., Zimmerman N.J., Lucchini R.G. (2012). Heavy metals in soil and salad in the proximity of historical ferroalloy emission. J. Environ. Prot..

[56] Al-Salama Y.J. (2013). The use of neutron activation analysis technique to estimate some heavy metals in soil and plants irrigated with wastewater. R.J. Aleppo Univ. Basic Sci. Ser..

[57] Armelin M.J.A., Trevizam A.R., Muraoka T., Silva M.L.S., Saiki M., Maihara V.A. (2011). Instrumental neutron activation analysis applied to multielement determination in a variety of lettuce grown in a contaminated soil and treated with phosphate. 3rd-INCC.

[58] Pacheco A.M.G., Freitas M.C., Ventura M.G., Dionısio I., Ermakova E. (2006). Chemical elements in common vegetable components of Portuguese diets, determined by k0-INAA. Nucl. Instrum. Methods Phys. Res. A.

[59] Ittipongse A., Fungklin R. (2016). Determine of heavy metal contents in fresh vegetable by using nuclear activation analysis technique. SNRU J. Sci. Technol..

[60] Freitas M.C., Pacheco A.M.G., Bacchi M.A., Dionísio I., Landsberger S., Braisted J., Fernandes E.A.N. (2008). Compton suppression instrumental neutron activation analysis performance in determining trace- and minor-element contents in foodstuff. J. Radioanal. Nucl. Chem..

[61] Alsayed E.M., Elqusy N.O. (2018). Heavy metals uptake and translocation by lettuce and spinach grown on a metal-contaminated soil. J. Soil Sci. Plant Nutr..

[62] Adu A.A., Aderinola O.J., Kusemiju V. (2012). Heavy metals concentration in garden lettuce (*Lactuca sativa* L.) grown along Badagry expressway, Lagos, Nigeria. Transnatl. J. Sci. Technol..

[63] Boamponsem G.A., Kumi M., Debrah I. (2012). Heavy metals accumulation in cabbage, lettuce and carrot irrigated with wastewater from Nagodi mining site in Ghana. Int. J. Sci. Technol. Res..

[64] Seka Yapoga J., Yapo Ossey B., Yapi Dopé A.C. (2015). Heavy metals contamination in *Lactuca sativa* L. (lettuce) from two agricultural sites of Abidjan. Int. J. Pure Appl. Sci. Technol..

[65] Lichtenthaler H.K., Buschmann C. (2001). Current Protocols in Food Analytical Chemistry (Units: F4.3.1–F4.3.8).

[66] Ivanova V., Stefova M., Chinnici F. (2010). Determination of the polyphenol contents in Macedonian grapes and wines by standardized spectrophotometric methods. J. Serb. Chem. Soc..

[67] Brand-Williams W., Cuvelier M.E., Berset C. (1995). Use of a free radical method to evaluate antioxidant activity. LWT-Food Sci. Technol..

